# Supplementary material for: Dysregulation of DNA Methylation and Epigenetic Clocks in Prostate Cancer among Puerto Rican Men
Source: Biomolecules. 2021 Dec 21;12(1):2. doi: 10.3390/biom12010002 (PMC8773891; doi:10.3390/biom12010002)
Supplement: Supplementary file 1 [file biomolecules-12-00002-s001.zip › biomolecules-1438489-supplementary.pdf]

Table S1. Differentially methylated genes between aggressive (high-risk) and indolent (low-risk) prostate tumors in Puerto Rican men with prostate cancer.

number	ProbeId	Gene Symbol	p-value	Mean Difference	Mean (Low)	Mean (High)
1	cg05013909	PRDM16	0.000758917	0.113	0.217	0.330
2	cg10143426	TP73	0.000874793	-0.113	0.230	0.117
3	cg17163404	UBR4	0.000514859	-0.127	0.602	0.474
4	cg08729059	ZNF593	0.000854296	0.072	0.303	0.374
5	cg02833367	PTP4A2	0.000341483	-0.060	0.715	0.655
6	cg19494946	BMP8A	0.000637902	-0.092	0.272	0.179
7	cg23682160	CTPS1	0.000657912	-0.124	0.390	0.266
8	cg06094523	SLC2A1	0.000862398	-0.073	0.458	0.385
9	cg13393721	MPL	0.000599594	0.090	0.124	0.214
10	cg00023154	RNF220	0.000297992	-0.103	0.205	0.102
11	cg05655221	STIL	0.00043066	-0.073	0.375	0.302
12	cg02904015	RABGGTB	0.000126125	0.093	0.126	0.220
13	cg17405164	ST6GALNAC5	0.000768965	-0.102	0.627	0.525
14	cg09499257	CCDC18	0.000465526	-0.092	0.687	0.594
15	cg06100373	CCDC18	0.000524264	-0.077	0.383	0.306
16	cg02472525	PALMD	0.000942609	-0.056	0.768	0.712
17	cg13876809	GPR88	0.000136333	-0.052	0.788	0.736
18	cg01435515	ZNF687	0.000350501	-0.102	0.311	0.209
19	cg01474866	CEP350	0.000309206	-0.080	0.398	0.318
20	cg18126097	NR5A2	0.000727335	-0.099	0.546	0.447
21	cg21218864	RASSF5	0.000830803	-0.100	0.355	0.256
22	cg22871559	RASSF5	0.000454232	-0.087	0.241	0.154
23	cg19405931	GPATCH2	0.000342785	-0.083	0.689	0.606
24	cg14378539	MIR548F3	0.000720107	0.067	0.788	0.855
25	cg14247594	TP53BP2	0.000977266	-0.096	0.622	0.526
26	cg15975832	TP53BP2	0.000777486	-0.098	0.475	0.377
27	cg10475729	STRN	0.000910539	-0.084	0.679	0.595
28	cg15211026	SPRED2	4.32E-05	-0.284	0.587	0.303
29	cg14551984	SGOL2	0.000825851	-0.083	0.425	0.342
30	cg07227024	ALS2CR12	0.000847484	-0.177	0.387	0.211
31	cg18899117	PARD3B	0.000330358	-0.057	0.778	0.721
32	cg03556763	ERBB4	0.000942874	-0.055	0.756	0.701
33	cg00007809	SEPT2	0.000472597	-0.076	0.640	0.563
34	cg10009111	TRNT1	0.000515945	-0.115	0.528	0.413
35	cg26986369	SRGAP3	0.000374609	-0.115	0.772	0.657
36	cg08525922	VGLL4	0.000137804	-0.117	0.433	0.315
37	cg00610238	CMC1	0.000353204	-0.057	0.802	0.745

38	cg18353563	ABTB1	0.000193826	-0.116	0.264	0.148
39	cg16141378	LOC729375	0.00056279	-0.051	0.132	0.082
40	cg11941811	TMEM108	1.12E-05	0.229	0.454	0.683
41	cg15048832	TSC22D2	0.000787958	-0.079	0.259	0.180
42	cg25296465	MUC20	0.000957367	-0.093	0.709	0.616
43	cg14724298	LNX1	0.000526647	-0.064	0.771	0.707
44	cg15127930	YTHDC1	0.000802867	-0.050	0.797	0.746
45	cg12677522	ZNF827	0.000746014	-0.086	0.674	0.588
46	cg05840230	TENM3	0.00055066	-0.122	0.508	0.387
47	cg16253976	ADCY2	0.000390071	-0.080	0.538	0.458
48	cg03661461	OSMR-AS1	0.000558618	-0.113	0.399	0.286
49	cg13566919	GZMA	0.000148831	-0.122	0.399	0.277
50	ch.5.1443044F	AGGF1	0.000342593	0.050	0.065	0.115
51	cg11442606	CDC42SE2	0.000524379	-0.063	0.797	0.734
52	cg27513482	FSTL4	0.000741069	-0.093	0.422	0.328
53	cg20518045	SPOCK1	0.000301381	-0.080	0.766	0.686
54	cg18771857	MATR3	0.000467242	-0.080	0.171	0.091
55	cg14710984	MGC29506	0.000185415	-0.163	0.725	0.562
56	cg08736680	MGC29506	0.000464954	-0.101	0.491	0.391
57	cg15263821	FAM65B	0.000786055	-0.063	0.785	0.721
58	cg21176130	HLA-E	8.56E-05	-0.109	0.222	0.113
59	cg09556042	HLA-C	0.000140772	-0.168	0.256	0.088
60	cg11917734	HLA-C	0.000124752	-0.152	0.214	0.062
61	cg11574174	HLA-C	0.000485259	-0.204	0.450	0.246
62	cg01521131	HLA-C	0.00064565	-0.105	0.280	0.174
63	cg11867651	HLA-C	1.46E-05	-0.173	0.309	0.135
64	cg19732154	GPSM3	0.000882858	-0.117	0.249	0.133
65	cg16513215	GPSM3	0.000811731	-0.148	0.419	0.271
66	cg26053840	GLO1	0.000813032	-0.110	0.507	0.397
67	cg14841847	LACE1	0.000425888	-0.072	0.289	0.218
68	cg11769349	KIAA1908	0.000881068	-0.164	0.469	0.306
69	cg23285459	GNA12	0.000324891	0.121	0.597	0.718
70	cg25961123	RAPGEF5	0.00061486	-0.052	0.817	0.765
71	cg10320911	BMPER	0.00047276	0.124	0.726	0.850
72	cg16874554	LOC100506725	8.72E-05	0.057	0.149	0.206
73	cg07976112	H2AFV	0.000764895	-0.108	0.356	0.248
74	cg12573289	CCM2	0.00048533	-0.076	0.553	0.477
75	cg27048140	ATP5J2	0.000608847	-0.089	0.627	0.538
76	cg22438280	COG5	0.000884102	-0.109	0.572	0.463
77	cg02345865	MKLN1	0.000693312	-0.062	0.770	0.708
78	cg17611936	PRKAG2	0.000761281	0.218	0.428	0.646
79	cg18857769	KMT2C	0.000106389	-0.091	0.461	0.370

80	cg08803663	FLJ10661	0.000315018	-0.056	0.133	0.078
81	cg23957763	DEFB134	0.00061027	-0.105	0.527	0.422
82	cg00495428	ELAVL2	0.000730612	-0.101	0.638	0.537
83	cg02522587	MOB3B	0.000993337	-0.100	0.560	0.460
84	cg14267542	KIAA0649	4.61E-05	-0.113	0.309	0.197
85	cg10331073	APBB1IP	0.000696745	0.094	0.166	0.260
86	cg06420074	CCNY	0.000123892	0.082	0.268	0.350
87	cg22174486	HRAS	0.000186634	-0.112	0.370	0.258
88	cg02030652	BRSK2	1.27E-05	-0.096	0.254	0.157
89	cg00117796	TEAD1	0.000389872	-0.106	0.386	0.280
90	cg10987969	INPPL1	1.10E-05	-0.066	0.430	0.365
91	cg15279866	ARAP1	0.000612813	-0.075	0.665	0.590
92	cg10064339	UCP2	0.000485493	-0.139	0.233	0.094
93	cg09458566	SIK3	0.000199561	-0.114	0.476	0.362
94	cg22997768	TAS2R14	0.000695507	-0.054	0.717	0.663
95	cg12253142	LRP6	0.00049211	-0.105	0.487	0.382
96	cg12254405	MANSC1	0.000815639	-0.116	0.583	0.466
97	cg05573680	HTR7P1	0.000562975	0.093	0.515	0.609
98	cg16789995	HEBP1	0.000253051	0.127	0.426	0.553
99	cg21585975	PLEKHA5	0.000588635	-0.095	0.526	0.431
100	cg07938212	BICD1	0.000347401	-0.102	0.425	0.323
101	cg19293650	KIF21A	0.000943484	-0.071	0.394	0.323
102	cg00280812	KRT80	0.000793018	-0.118	0.560	0.442
103	cg20519295	LOC102724421	0.00086942	-0.088	0.688	0.600
104	cg09711238	IFNG	0.000395939	-0.080	0.371	0.291
105	cg01576496	PPP1R12A	0.000779914	-0.120	0.641	0.521
106	cg18027157	IGF1	0.000950191	-0.100	0.541	0.441
107	cg20074271	C12orf42	0.00056127	-0.135	0.566	0.431
108	cg23793186	GIT2	0.000727405	-0.112	0.565	0.452
109	cg16412776	TCTN1	0.000371664	-0.082	0.642	0.560
110	cg06331841	DGKH	0.000799419	-0.096	0.524	0.428
111	cg09953995	LMO7	0.000702941	-0.069	0.805	0.736
112	cg23664361	RNASE13	0.00061225	0.081	0.684	0.765
113	cg27050584	KIAA0391	0.000354383	-0.091	0.390	0.300
114	cg00771309	TMEM260	0.000488084	-0.100	0.422	0.322
115	cg24684798	RGS6	0.000855013	0.077	0.272	0.349
116	cg09306332	GRAMD2	0.000790499	-0.088	0.716	0.627
117	cg02127888	CRAMP1L	0.000853591	-0.096	0.399	0.303
118	cg00406621	BOLA2	0.000850719	-0.072	0.187	0.115
119	cg25206919	TRIM72	0.000836752	-0.103	0.216	0.112
120	cg04060128	TGFB1I1	0.000847413	-0.141	0.686	0.545
121	cg20630240	DYNC1LI2	0.000730542	0.092	0.375	0.466

122	cg24867981	LOC101927817	0.000531214	-0.102	0.590	0.488
123	cg23630423	RTN4RL1	0.000783657	0.051	0.107	0.159
124	cg23571857	XAF1	0.000459537	0.086	0.571	0.657
125	cg05949005	SLC2A4	0.000810558	-0.086	0.171	0.085
126	cg13895343	SARM1	0.000360462	-0.116	0.676	0.561
127	cg12139369	CDC42EP4	0.000951048	-0.084	0.371	0.287
128	cg08267319	LOC92659	4.95E-05	-0.147	0.483	0.336
129	cg10801102	DUS1L	0.000899202	-0.114	0.228	0.115
130	cg02944903	SMCHD1	0.000882919	-0.104	0.507	0.402
131	cg15184164	CEP192	0.000847067	-0.087	0.407	0.320
132	cg24736345	GNA11	0.000952583	0.086	0.737	0.822
133	cg02584678	NOTCH3	0.00097665	-0.134	0.458	0.324
134	cg11013011	HAUS8	0.00046855	-0.110	0.204	0.094
135	cg09846076	ZNF540	0.000832576	-0.072	0.174	0.102
136	cg03975694	ZNF540	4.53E-05	-0.187	0.336	0.149
137	cg02798280	MAP4K1	0.000636099	0.099	0.303	0.402
138	cg09565670	RINL	0.000379724	-0.107	0.519	0.412
139	cg12978040	SAPS1	0.000622124	-0.078	0.336	0.258
140	cg08335200	RIN2	0.000138318	-0.132	0.399	0.267
141	cg26232247	RIN2	0.000947988	-0.106	0.287	0.181
142	cg26396492	RIN2	0.000578362	-0.119	0.373	0.254
143	cg09035925	RIN2	0.000311899	-0.135	0.402	0.268
144	cg04466743	RIN2	0.0005589	-0.113	0.516	0.404
145	cg05826429	TP53INP2	0.000718904	0.074	0.247	0.321
146	cg11392297	UCKL1	0.000467598	-0.079	0.637	0.558
147	cg05480278	KRTAP6-1	0.000266004	-0.070	0.382	0.312
148	cg02289722	APOL3	0.000878568	0.068	0.651	0.719
149	cg04880355	MIR1249	0.000866532	-0.087	0.830	0.743
150	cg01990106	NLGN4X	0.000462668	-0.133	0.612	0.479
151	cg13826622	MID1	0.00077462	-0.102	0.686	0.584
152	cg10782870	IL1RAPL1	0.000736849	-0.100	0.670	0.570
153	cg16831427	AWAT2	0.000755782	-0.111	0.514	0.403
154	cg12100307	P2RY4	0.000783116	-0.060	0.652	0.592
